# Nonequilibrium thermodynamics and mitochondrial protein content predict insulin sensitivity and fuel selection during exercise in human skeletal muscle

**DOI:** 10.3389/fphys.2023.1208186

**Published:** 2023-07-07

**Authors:** Rocio Zapata Bustos, Dawn K. Coletta, Jean-Philippe Galons, Lisa B. Davidson, Paul R. Langlais, Janet L. Funk, Wayne T. Willis, Lawrence J. Mandarino

**Affiliations:** ^1^ Division of Endocrinology, Department of Medicine, The University of Arizona, Tucson, AZ, United States; ^2^ Center for Disparities in Diabetes, Obesity, and Metabolism, University of Arizona, Tucson, AZ, United States; ^3^ Department of Physiology, The University of Arizona, Tucson, AZ, United States; ^4^ Department of Medical Imaging, The University of Arizona, Tucson, AZ, United States

**Keywords:** skeletal muscle, 31 P-magnetic resonance spectroscopy, mitochondria, exercise, insulin sensitivity, fuel selection

## Abstract

**Introduction:** Many investigators have attempted to define the molecular nature of changes responsible for insulin resistance in muscle, but a molecular approach may not consider the overall physiological context of muscle. Because the energetic state of ATP (ΔG_ATP_) could affect the rate of insulin-stimulated, energy-consuming processes, the present study was undertaken to determine whether the thermodynamic state of skeletal muscle can partially explain insulin sensitivity and fuel selection independently of molecular changes.

**Methods:** ^31^P-MRS was used with glucose clamps, exercise studies, muscle biopsies and proteomics to measure insulin sensitivity, thermodynamic variables, mitochondrial protein content, and aerobic capacity in 16 volunteers.

**Results:** After showing calibrated ^31^P-MRS measurements conformed to a linear electrical circuit model of muscle nonequilibrium thermodynamics, we used these measurements in multiple stepwise regression against rates of insulin-stimulated glucose disposal and fuel oxidation. Multiple linear regression analyses showed 53% of the variance in insulin sensitivity was explained by 1) VO_2max_ (*p* = 0.001) and the 2) slope of the relationship of ΔG_ATP_ with the rate of oxidative phosphorylation (*p* = 0.007). This slope represents conductance in the linear model (functional content of mitochondria). Mitochondrial protein content from proteomics was an independent predictor of fractional fat oxidation during mild exercise (R^2^ = 0.55, *p* = 0.001).

**Conclusion:** Higher mitochondrial functional content is related to the ability of skeletal muscle to maintain a greater ΔG_ATP_, which may lead to faster rates of insulin-stimulated processes. Mitochondrial protein content *per se* can explain fractional fat oxidation during mild exercise.

## Introduction

Insulin resistance in skeletal muscle is a long-recognized hallmark feature of type 2 diabetes mellitus and obesity. Although type 2 diabetes generally only occurs when pancreatic beta cells are incapable of secreting enough insulin to maintain normoglycemia, this most often occurs in the setting of insulin resistance (DeFronzo et al., 2015). Numerous investigators using *in vivo* approaches in humans, rodent models, or *in vitro* experiments have attempted to define the molecular nature of the changes responsible for insulin resistance in skeletal muscle. Studies too numerous to cite completely have described molecular defects in insulin as action and fuel selection (Kahn and Cushman, 1985; Mandarino et al., 1986; Kelley and Mandarino, 1990; Cusi et al., 2000; Kelley and Mandarino, 2000; Patti et al., 2003). Despite learning a great deal of molecular physiology from these studies, this approach usually does not consider the overall physiological context of the skeletal muscle in which such defects may be found.

A different approach to understanding insulin action in skeletal muscle is to start from first principles, in this case from the observation that the energy state of resting and mildly exercising skeletal muscle is held far from equilibrium by the action of creatine kinase. Nonequilibrium thermodynamic theory arises from properties of thermodynamic equilibrium and can explain the mechanism and consequences of the extent to which skeletal muscle is able to maintain a high energy state when energy demand increases. In skeletal muscle, this energy state can be estimated as the energy made available by ATP hydrolysis, often termed ΔG_ATP_. Mitochondria maintain this energy at rest and defend it from falling too steeply under conditions of energy demand, such as muscle contraction or other energy consuming processes, which might include insulin action. Insulin at physiologic concentrations measurably increases oxygen consumption in leg muscle, demonstrating an increase in energy demand (Kelley and Mandarino, 1990; Kelley et al., 1992; Mandarino et al., 1993; Mandarino et al., 1996).

It is well known that a higher ATP energy state is linearly related to power output of the contractile apparatus (Jeneson et al., 1995; Westerhoff et al., 1995). This relationship would apply not only to muscle contraction but also to the many processes that require movement within a cell, and we recently proposed that this principle could apply to transport processes like insulin-stimulated, kinesin-powered movement of GLUT4 vesicles along microtubules (Mandarino and Willis, 2023) due to similarities between kinesins and myosins (Vale and Milligan, 2000). Insulin sensitivity, therefore, could be a function of the ability of the cell to maintain the energy state of ATP, which depends mitochondrial functional capacity (Paganini et al., 1997; Glancy et al., 2008).

Thermodynamic variables can be measured *in vivo* in human skeletal muscle using calibrated ^31^P-MRS at rest or during recovery from muscle contraction (Kemp et al., 2007; Kemp et al., 2015; Sleigh et al., 2016). ^31^P-MRS allows estimation of the free energy of ATP noninvasively and continuously at rest, during exercise, and during recovery from exercise. ^31^P-MRS also can be used to estimate the rate of ATP synthesis (J_ATP_) during recovery from exercise by monitoring the recovery of the concentration of phosphocreatine (Kemp et al., 2007; Kemp et al., 2015; Sleigh et al., 2016). PCr recovery from exercise follows a monoexponential time course, allowing estimation of the time constant parameter *τ* (Kemp et al., 2007; Kemp et al., 2015; Sleigh et al., 2016). However, ^31^P-MRS does not provide concentrations of energy phosphates unless spectra are calibrated to a standard, and unphosphorylated creatine is not visible. Because absolute concentrations of energy phosphate compounds such as PCr, ATP, ADP, as well as phosphate (Pi) are required to estimate thermodynamic variables, muscle biopsies at rest can be performed to assay [ATP], [PCr], and [Pi], allowing calculation of [ADP], the concentration of which is too low to be observed with MRS. Total creatine also can be assayed in muscle biopsies, providing all the absolute concentrations needed to calculate the energy of ATP (ΔG_ATP_) and the rate of ATP synthesis (J_ATP_). In skeletal muscle, mitochondria are responsible for most ATP production and maintain a high energy state of ATP. Increased ATP demand and the increased rate of ATP hydrolysis cause ΔG_ATP_ to fall in a nearly linear manner (Jeneson et al., 1995; Westerhoff et al., 1995). In response, J_ATP_ rises. The slope of this relationship depends on the cellular content of functional mitochondria (Dudley et al., 1987). Higher mitochondrial functional content enables better maintenance of ΔG_ATP_ (Paganini et al., 1997; Glancy et al., 2008).

A linear electrical circuit analog of the nearly linear kinetics of the relationship between the rate of oxidative phosphorylation and the energy from ATP has been used for over 30 years to simplify nonequilibrium thermodynamic considerations in skeletal muscle during non-steady state conditions when ΔG_ATP_ is changing (Meyer, 1989). There is theoretical and experimental evidence indicating that this model predicts linear relationships between 1) ΔG_ATP_ and J_ATP_ (the “force-flow” relationship), and 2) between the slope of the force-flow relationship and the functional mitochondrial content or “conductance” of the system. Establishment of these linear relationships is a key to validating this model in the context of the questions posed here. Conductance closely reflects mitochondrial functional content (Paganini et al., 1997; Glancy et al., 2008), while capacitance is proportional to the total creatine concentration, and affects the time course of changes during non-steady state conditions (Meyer, 1989). Finally, *τ*, the time constant of recovery of phosphocreatine after exercise, is the third variable needed to completely determine this model linear system. All of these parameters were estimated in the present study using a combination of ^31^P-MRS and biochemical assays in muscle biopsies. So, these studies can provide complete and detailed information about skeletal muscle nonequilibrium thermodynamics and the functional content of mitochondria needed to maintain a high energy state of the cell, which will drive energy-consuming processes more robustly (Jeneson et al., 1995). The reader interested in a more detailed theoretical treatment of calculations of thermodynamic parameters and derivation of the non-steady state treatment of nonequilibrium thermodynamics, including the meaning of the theoretical constructs of conductance and capacitance can refer to the Supplementary Material (Davis and Davis-Van Thienen, 1989; De Saedeleer and Marechal, 1984; Glancy et al., 2013; Golding et al., 1995; Harris et al., 1992; Hasselbach and Oetliker, 1983; Jeneson et al., 2009; Kushmerick, 1998; Masuda et al., 1990; Nicholls and Ferguson, 2013; Pate et al., 1998; Rottenberg, 1973; Walter et al., 1997; Westerhoff et al., 1981; Westerhoff and Van Damm, 1987; Willis et al., 2016).

We refer to mitochondrial protein abundance, determined using proteomics, as mitochondrial protein content, a biochemical measure of the abundance of all mitochondrial proteins, to distinguish it from mitochondrial functional content, which can differ from mitochondrial protein content to the extent that molecular changes in mitochondria may either increase or decrease the ability of a given mass of mitochondria to influence the rate of oxidative phosphorylation. The functional advantages of both higher mitochondrial protein and mitochondrial functional content can be seen in muscle adapted to endurance exercise, where there is a better ability to maintain the energy of ATP (Paganini et al., 1997), fuel selection that favors lipid over carbohydrate oxidation (Holloszy and Coyle, 1984; Helge et al., 2007), and better fatigue resistance (Fitts et al., 1975).

Many processes activated by insulin, such as GLUT4 translocation, require energy from ATP hydrolysis and therefore would have rates that are dependent on the energy of ATP. Compared to our knowledge of muscle contraction, we know much less about the impact of skeletal muscle nonequilibrium thermodynamics and mitochondrial functional content on skeletal muscle metabolism at rest or during insulin stimulation, especially in the setting of insulin resistance. Therefore, it is important to consider whether functional mitochondrial content and the capacity to maintain a higher ATP energy influences the rates of insulin-dependent processes, as recently conjectured (Mandarino and Willis, 2023). The primary aim of this investigation therefore was to use ^31^P-MRS, muscle biopsies, euglycemic clamps, and exercise tests in the context of the linear model to explore whether and to what extent nonequilibrium thermodynamic parameters and skeletal muscle mitochondrial functional content are related to insulin sensitivity and fuel selection (Mandarino and Willis, 2023). Finally, we measured VO_2max_ because of its well-described positive association with insulin sensitivity that likely is independent of mitochondrial content and more dependent on the cardiorespiratory system and delivery of oxygen to muscle and the capacity for muscle blood flow (Lundby et al., 2017). Linear models were used to determine which of these variables independently predict insulin sensitivity determined using a euglycemic clamp or fuel selection during mild exercise (Barakati et al., 2022).

## Materials and methods

### Participants and study design

**FIGURE 1:**
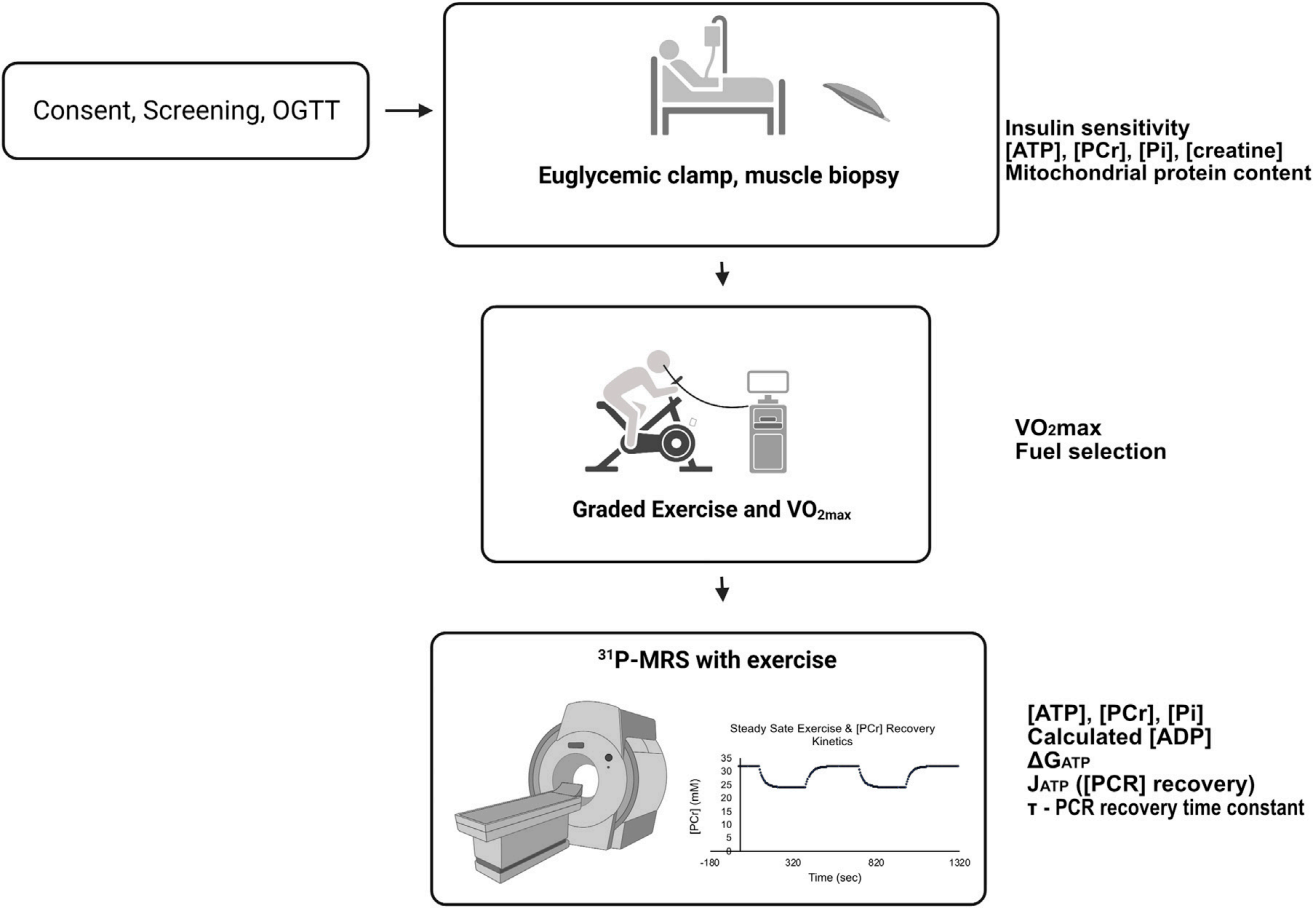
Participants first had a consent and screening visit, followed by a euglycemic, hyperinsulinemic clamp experiment with resting muscle biopsy, submaximal and maximal cycle ergometer exercise studies with indirect calorimetry. Finally, all participants underwent ^31^P-MRS studies that included resting and knee-extension exercise periods. For details, see Methods. The experiments were conducted on separate days approximately 1 week apart. Variables and measurements are listed adjacent to the study day from which they were derived.

Sixteen volunteers were screened with a history and physical examination, laboratory measurements, body composition (bioimpedance), an ECG, and a 75 g oral glucose tolerance test. Participants were not taking medications or supplements that affect glucose metabolism and were instructed to maintain their usual diet and not to engage in exercise 48 h before any testing. Volunteers were selected to have a wide range of body composition, aerobic capacity, and insulin sensitivity to reveal relationships among variables and detect the predictive value of thermodynamic, kinetic, and other variables. The design of the studies is given in Figure 1. All participants completed four study visits; a consent and screening examination, a euglycemic clamp experiment with muscle biopsy taken under resting, postabsorptive conditions, a graded cycle ergometry exercise test with indirect calorimetry and subsequent VO_2max_ determination, and a^31^P-MRS study with two periods of rest, leg extension exercise, and recovery from exercise, all conducted in the magnet. All studies were approved by the University of Arizona Institutional Review Board.

### Euglycemic, hyperinsulinemic clamps, and muscle biopsies

All volunteers underwent a euglycemic, hyperinsulinemic clamp with a resting, basal muscle biopsy, starting at 7–8 a.m. after an overnight fast (Cusi et al., 2000). Isotopically labeled glucose (6,6-dideuteroglucose, Cambridge Laboratories) was used to trace glucose metabolism (Lefort et al., 2010), and steady state conditions were assumed for calculating the rates of glucose metabolism. Participants had a percutaneous needle biopsy of the *vastus lateralis* muscle taken under basal conditions 1 h before starting an insulin infusion at a rate of 80 mU·m^−2^·min^−1^. Biopsies were frozen in liquid nitrogen before analysis (Cusi et al., 2000; Lefort et al., 2010). These biopsies were used to calibrate energy phosphates and total creatine.

### Exercise testing

On another day, at least 1 week separated from the glucose clamp, gas exchange measurements were made at rest and during progressive cycle ergometer exercise using a Parvo Medics metabolic measurement system (TrueOne 2400, Parvo Medics, Salt Lake City, Utah) (Barakati et al., 2022). This system uses gas measurements from a mixing chamber with sampling four times per minute. After catheter placement for blood sampling, subjects rested on the cycle for 6 min and then exercised at power outputs of 15, 30, and 45 W for 6 minutes each. VO_2_ and VCO_2_ values at rest and during exercise were used to calculate ΔVO_2_ and ΔVCO_2_ during exercise. Delta values were used to calculate a Respiratory Exchange Ratio due to working muscle (ΔRER = ΔVCO_2_/ΔVO_2_). ΔRER estimates the oxidative metabolism of muscle performing mild exercise and we have validated this method previously for walking (Willis et al., 2005) and mild cycle ergometer exercise (Barakati et al., 2022). This was followed continuously by a ramp protocol to determine VO_2max_.

### Calibrated ^31^P magnetic resonance spectroscopy (MRS)

On a separate day, ^31^P- MRS was performed on a research-dedicated 3.0 T Skyra scanner (Siemens, Erlangen, Germany). For ^31^P data, a 60-mm-diameter coil (double tuned for ^31^P and ^1^H) was positioned over the *vastus lateralis*. From the dimension of the coil and the size/geometry of a typical upper leg most of the signal in the unlocalized ^31^P-MRS measurements originates in the *vastus* (43). A ^1^H-MR image was obtained (using the body coil of the 3.0 T system for transmission and the ^31^P/1H surface coil for detection) to assure correct coil position before acquiring ^31^P spectra. ^31^P data were acquired using free induction decay spectroscopy (spectral width = 2,000 Hz, 1,024 data points) (fully relaxed conditions, flip angle = 90^o^, 20 s interpulse delay, 8 averages). Knee extension exercise was performed in the magnet using a weighted bag system (Sleigh et al., 2016). Bag weight was calibrated to individual exercise characteristics. Spectra were fitted in the time domain using a nonlinear least squares algorithm, jMRUI (Vanhamme et al., 1997) and fully relaxed spectra were used to quantify ATP, PCr, and Pi peak areas. Energy phosphate peak areas were calibrated to total adenylate content of muscle biopsies, assuming >99% of adenylates are present as ATP at the equilibrium established by creatine kinase. We ensured this by adding a phosphorylating system prior to luciferase detection of ATP (Lust et al., 1981; Passonneau and Lowry, 1993). Total creatine (TCr) concentration was assayed in muscle biopsies (Passonneau and Lowry, 1993). [ADP] was calculated using the creatine kinase equilibrium equation. ΔG_ATP_ values and J_ATP_ were calculated as described (Meyerspeer et al., 2020); individual time constants of recovery of PCr after exercise, *τ*, were derived by fitting recovery kinetics to a monoexponential function. This monoexponential function also yields the rate constant k (units of min^−1^), which is used to calculate the instantaneous rate of oxidative phosphorylation (J_ATP_), assuming J_ATP_ = the rate of resynthesis of PCr after exercise ceases. Thus, J_ATP_ (mM/min) = k ([PCR]_ss_—[PCR]_t_), where [PCr]_ss_ is the concentration of PCr at rest and [PCr]_t_ is the concentration of PCr at each timepoint t during recovery from exercise.

### Proteomic assessment of mitochondrial protein content

Whole muscle (75 µg) lysates were resolved on 4%–20% gradient polyacrylamide gels, which were stained with Bio-Safe Coomassie G-250 Stain (#1610786; Biorad, Hercules, CA). Each lane was cut into 6 bands, which were excised and digested with trypsin. Samples were desalted and purified with C^18^ columns and evaporated and reconstituted in 30% acetonitrile/0.1% trifluoroacetic acid (TFA) immediately before mass spectrometry analysis. HPLC-ESI-MS/MS was performed in positive ion mode on a Thermo Scientific Orbitrap Fusion Lumos tribrid mass spectrometer fitted with an EASY-Spray Source (Thermo Scientific, San Jose, CA) (Parker et al., 2019). NanoLC was performed without a trap column using a Thermo Scientific UltiMate 3000 RSLCnano System with an EASY Spray C18 LC column (Thermo Scientific, 50 cm × 75 μm inner diameter, packed with PepMap RSLC C18 material, 2 μm, cat. #ES903); loading phase for 15 min at 0.300 μL/min; mobile phase, linear gradient of 1%–34% Buffer B in 119 min at 0.220 μL/min, followed by a step to 95% Buffer B over 4 min at 0.220 μL/min, hold 5 min at 0.250 μL/min, and then a step to 1% Buffer B over 5 min at 0.250 μL/min and a final hold for 10 min (total run 159 min); Buffer A = 0.1% FA/H_2_O; Buffer B = 0.1% FA in 80% ACN. Solvents were liquid chromatography mass spectrometry grade. Spectra were acquired using XCalibur, version 2.3 (Thermo Scientific). A “top speed” data-dependent MS/MS analysis was performed. Dynamic exclusion was enabled with a repeat count of 1, a repeat duration of 30 s, and an exclusion duration of 60 s. A whole muscle proteome was quantified by label-free analysis using Progenesis QI software (Nonlinear Dynamics/Waters, Milford, MA). Normalized peak areas of all proteins annotated to mitochondria were summed into an index of total mitochondrial protein abundance. Individual normalized peak areas of mitochondrial and total proteins are given in Supplementary Table S1. Citrate synthase activity was also assayed in homogenates of muscle biopsies (Srere, 1969). The mass spectrometry proteomics data have been deposited to the ProteomeXchange Consortium via the PRIDE partner repository with the dataset identifier PXD043032 and 10.6019/PXD043032.

### Theoretical considerations

Please see Supplementary Material for theoretical background, assumptions, and calculations for thermodynamic parameters such as ΔG_ATP_ as well as the linear electrical circuit model of nonequilibrium thermodynamics proposed by Meyer to explain the linear relationship of τ with mitochondrial functional content (“conductance”) and capacitance represented by TCr, or total creatine (Meyer, 1989).

### Analytical assays

Enrichment of deuterated glucose was determined by LC-MS (Lalia et al., 2016). Insulin was assayed using ELISA (Alpco, Salem, NH). Concentrations of total adenylates and creatine were assayed in muscle biopsies using modifications of published methods (Passonneau and Lowry, 1993) and expressed as a concentrations per volume muscle cell water.

### Calculations and statistics

Rates of glucose turnover were calculated using steady state equations (Debodo et al., 1963). Statistical comparisons were performed using t-tests and stepwise multiple linear regression removing the least significant variable. Pearson’s correlation coefficient was used to assess relationships between two variables, using one or two-tailed tests as appropriate. Monoexponential curve fitting for estimating τ from PCr recovery kinetics was performed using the Solver add-in for Excel.

## Results

### Characteristics

Sixteen people participated in this study (4 men, 12 women). Participant characteristics are given in Table 1. Four participants had untreated type 2 diabetes as determined using HbA1c (8.1% ± 1.0%) or fasting plasma glucose (157 ± 37 mg/dL), four others had prediabetes (HbA1c 6.0% ± 1.0%), and the remainder had normal glucose tolerance (HbA1c 5.4% ± 0.2%). Endogenous glucose production in the postabsorptive state was 3.46 ± 0.13 mg/(kg-FFM^·^min) and was completely suppressed during the insulin-infusion. Rates of basal and insulin-stimulated glucose metabolism are given in Table 1.

**TABLE 1:** Subject characteristics.

	Mean	SD
Age (years)	46.9	14.0
Weight (kg)	94.5	27.1
BMI (kg/m^2^)	33.4	9.4
Fat mass (kg)	38.3	17.3
Lean mass (kg)	56.3	12.9
Body Fat %	39.0	9.5
HbA1c, %	6.3	1.3
Fasting glucose (mg/dL)	109.4	34.1
Fasting insulin (µIU/mL)	12.3	9.1
Clamp insulin (µIU/mL)	197	23
Basal glucose disposal (mg^•^kg-FFM^−1•^min^−1^)	3.43	0.12
Clamp glucose disposal (mg^•^kg-FFM^−1•^min^−1^)	6.44	1.02
Basal endogenous glucose production (mg^•^kg-FFM^−1•^min^−1^)	3.32	0.12
Clamp endogenous glucose production (mg·kg-FFM^−1^·min^−1^)	−0.27	0.18
VO_2max_ (ml O_2_·kg-FFM^−1^·min^-1^)	32.9	11.2

Data are given as Mean ± Standard Deviation (SD). FFM, fat free mass.

### Calibrated 31P-MRS

**FIGURE 2:**
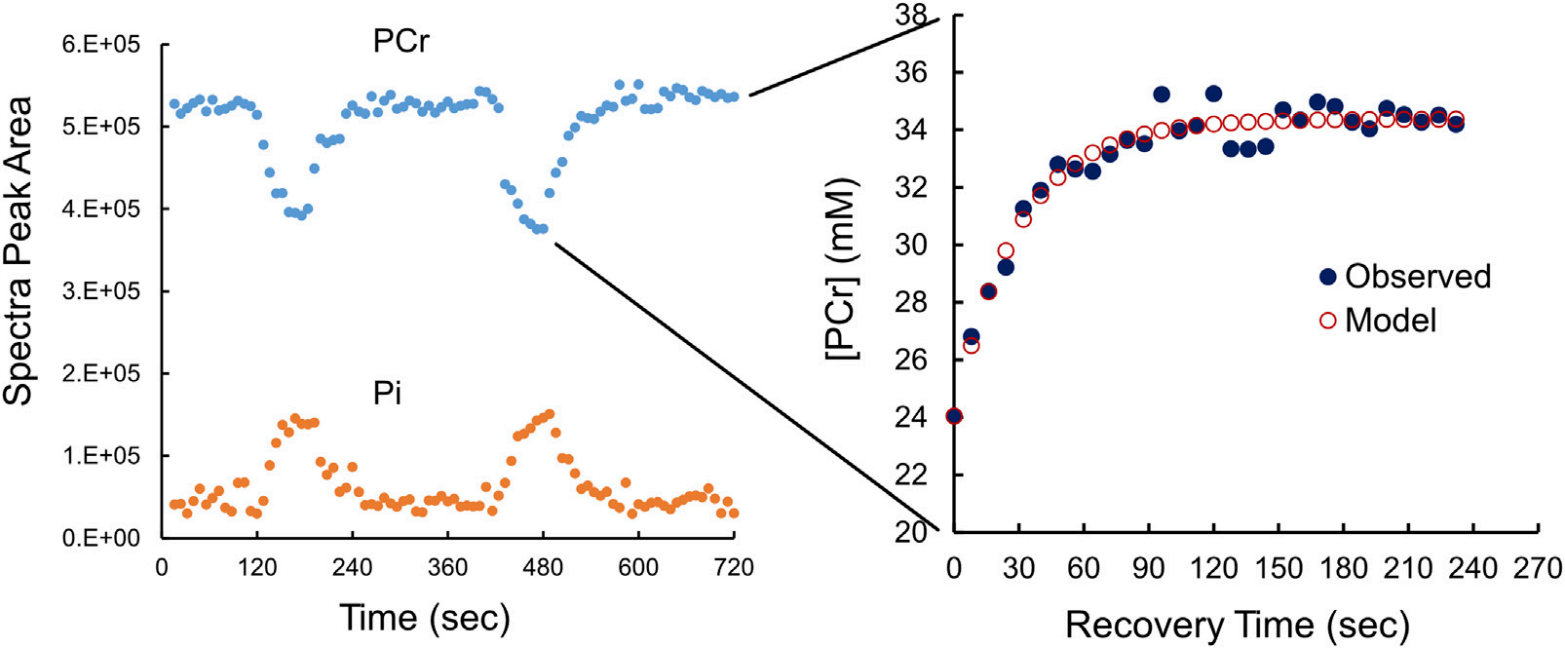
Phosphocreatine (PCr) and phosphate (Pi) kinetics during exercise and resting recovery monitored using ^31^P-MRS. The left panel, using uncalibrated ^31^P-MRS data (peak areas) shows the fall in [PCr] is matched by a rise in [Pi] during exercise. Each of two periods of resting recovery from exercise was fitted to a monoexponential function (see cutout) to derive τ, the time constant for recovery by calibrated [PCr] to a monoexponential function. The right panel shows calibrated [PCr] (black circles) with the fitted monoexponential function (red circles). τ is the time constant of the monoexponential function.

Calibrated ^31^P-MRS was used to measure [ATP], [PCr], and [Pi] at rest, during 1.0 min of knee extension exercise, and then 4.0 min of recovery Table 2; Figure 2. This rest-exercise-recovery protocol was performed twice while the participant was positioned in the magnet. Results of biochemical analyses of total adenylates (ATP) and TCr in muscle biopsies were used to calibrate ^31^P-MRS energy phosphate concentrations on an individual level. These concentrations are given at rest and at end of exercise in Table 2. All ^31^P-MRS-determined peak areas of PCr, ATP, and Pi were calibrated to [ATP] determined using biopsies taken under basal, resting conditions. Total adenylates, a good estimate of resting [ATP] in muscle (Connett, 1989), averaged 6.7 ± 0.2 mM in the biopsies while total creatine (TCr) was 38.5 ± 7.3 mM. Free creatine was calculated as the difference between TCr and [PCr] calibrated to [ATP] in each biopsy. [ADP] was calculated from calibrated values of energy phosphates continuously using the creatine kinase equilibrium equation (Supplementary Material) (Veech et al., 1979), rising from about 18.8 μM at rest to nearly 38 µM after exercise. As expected, [PCr] fell significantly by the end of each exercise period with a nearly equimolar concomitant rise in [Pi]; [Pi] rose from 2.79 at rest to 7.05 mM at end of exercise. Resting ΔG_ATP_ fell about 1 kcal/mol on average, while pH rose minimally with exercise.

**TABLE 2:** Skeletal muscle total creatine, phosphocreatine, ATP, ADP, pH, and ΔG_ATP_ at rest and end of exercise.

	Rest		End exercise				
	Mean	SD	Mean		SD		
Total Creatine (TCr, mM)	38.5	7.3	-		-		
Phosphocreatine (PCr, mM)	27.0	4.3	21.7**		4.2		
ATP (mM)	6.75	1.0	6.47		1.1		
ADP (µM)	18.8	6.8	37.7**		12.4		
Pi (mM)	2.79	0.60	7.05**		1.6		
PCr/TCr	0.71	0.10	0.57**		0.08		
pH	7.04	0.02	7.09**		0.03		
ΔG_ATP_ (kcal·mole^−1^)	−14.95	0.30	−13.82**		0.30		

Data are given as Mean ± Standard Deviation (SD), *n* = 16. [ADP] was calculated using the creatine kinase equilibrium equation. Energy phosphate spectra from.

^31^P-MRS, were calibrated to resting [ATP]. **p < 0.01 vs resting values. End exercise values for TCr, were assumed to be equal to resting values. End of exercise values are average of two exercise periods in the magnet.

A typical example, in duplicate, of ^31^P-MRS-determined changes in [PCr] and [Pi] from rest to end-exercise and then recovery is shown in Figure 2. The cutout in Figure 2 also shows the monoexponential recovery from end-exercise to rest that was used to estimate *τ*, the time constant of recovery. Observed values were fit to a monoexponential equation to derive values of *τ* in duplicate for each participant as exemplified in Figure 2. The average value for *τ* was 30.9 ± 6 s, with a coefficient of variation of about 13.6%.

### Conformation of data to the linear electrical circuit analog model of thermodynamics in healthy and insulin resistant skeletal muscle

**FIGURE 3:**
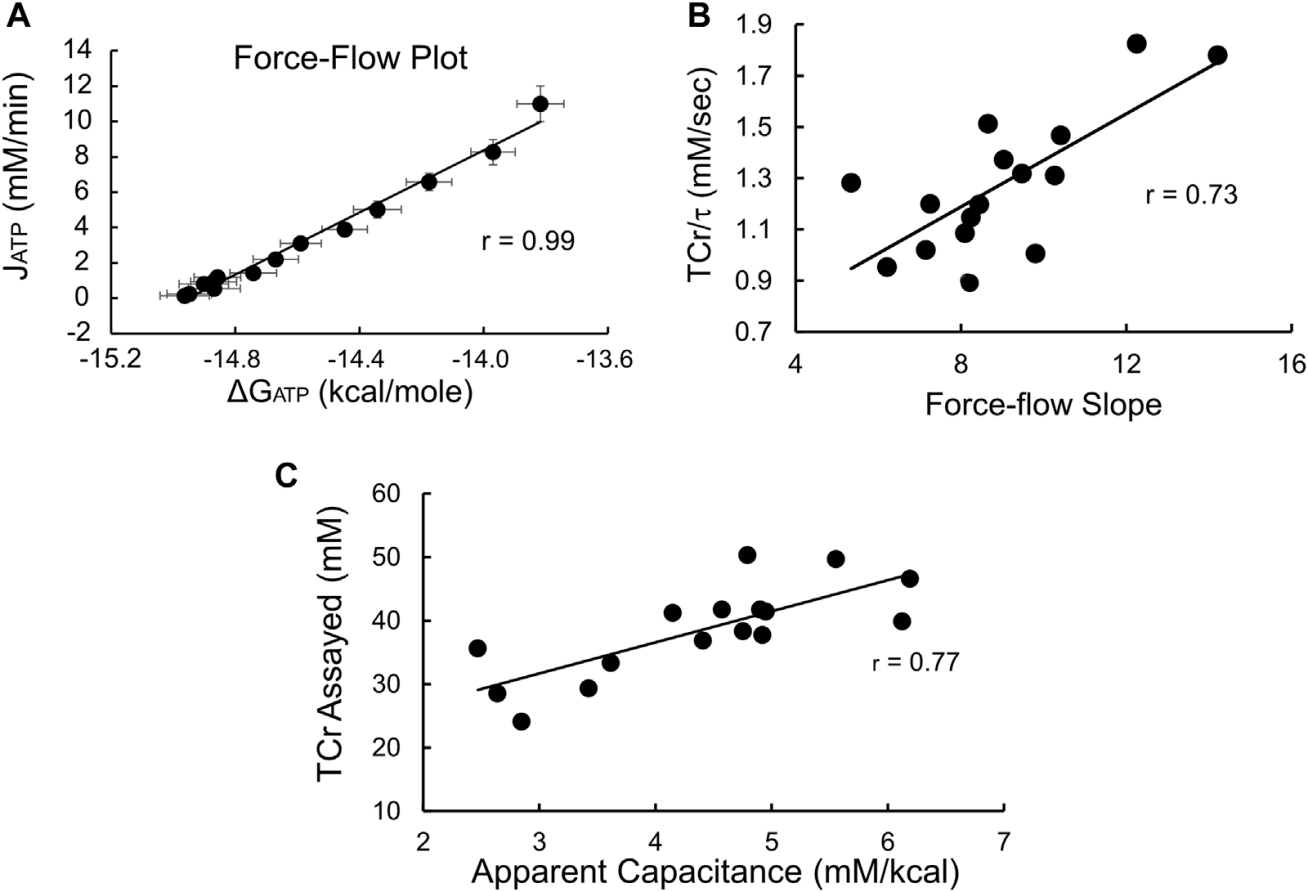
^31^P-MRS produces data that conforms to the electrical circuit analog model of skeletal muscle nonequilibrium thermodynamics. **(A)** Force-flow relationship between ΔG_ATP_ and the rate of oxidative phosphorylation (J_ATP_). Shown are average values ±SD (*n* = 16); **(B)** Slope of the ΔG_ATP_:J_ATP_ relationship vs. conductance, estimated as the ratio TCr/τ (see Supplementary Material). Both the ΔG_ATP_:J_ATP_ slope and TCr/τ are estimates of conductance, that is, mitochondrial functional content. The high degree of correlation confirms the consistency of our data with the linear model. The units of the **(C)** Apparent capacitance, calculated as the product of τ and conductance, is linearly related to the assayed value of TCr, providing additional evidence of the validity of the ^31^P-MRS measurements and confirming that biopsy-measured TCr is proportional to the capacitance term in the linear model.

During recovery from exercise estimates of the instantaneous values of both [PCr] andΔG_ATP_ were used to construct the expected near-linear relationship between ΔG_ATP_ and oxidative phosphorylation (J_ATP_), (Meyer, 1988; Meyer, 1989). As shown in Figure 3A, averaged values were highly linear (*r* = 0.99). In addition, the individual force-flow slopes averaged 0.98 ± 0.01, with the lowest correlation coefficient being 0.95, emphasizing the linearity of these relationships on an average and individual level. Likewise (Figure 3B), the individual slopes of the ΔG_ATP_ vs. J_ATP_ force-flow relationship were a significant (*p* < 0.01) linear predictor of TCr/τ, the “conductance” term of the Meyer model (Eq. 16, Supplementary Material), that is a measure of mitochondrial functional content (Paganini et al., 1997; Glancy et al., 2008). Finally, the apparent capacitance derived as a theoretical construct of the linear model (calculated as the product of τ and the force-flow slope, or conductance, see Supplementary Material) was linearly related to biopsy-measured TCr (Figure 3C), also as predicted by the linear model of nonequilibrium thermodynamics (Eq. 15, Supplementary Material). Taken together, these results indicate that these measurements can be used validly to assess effects on insulin sensitivity or fuel oxidation in the context of the linear model of nonequilibrium thermodynamics in muscle.

### Graded exercise, fuel selection, and VO_2max_

**FIGURE 4:**
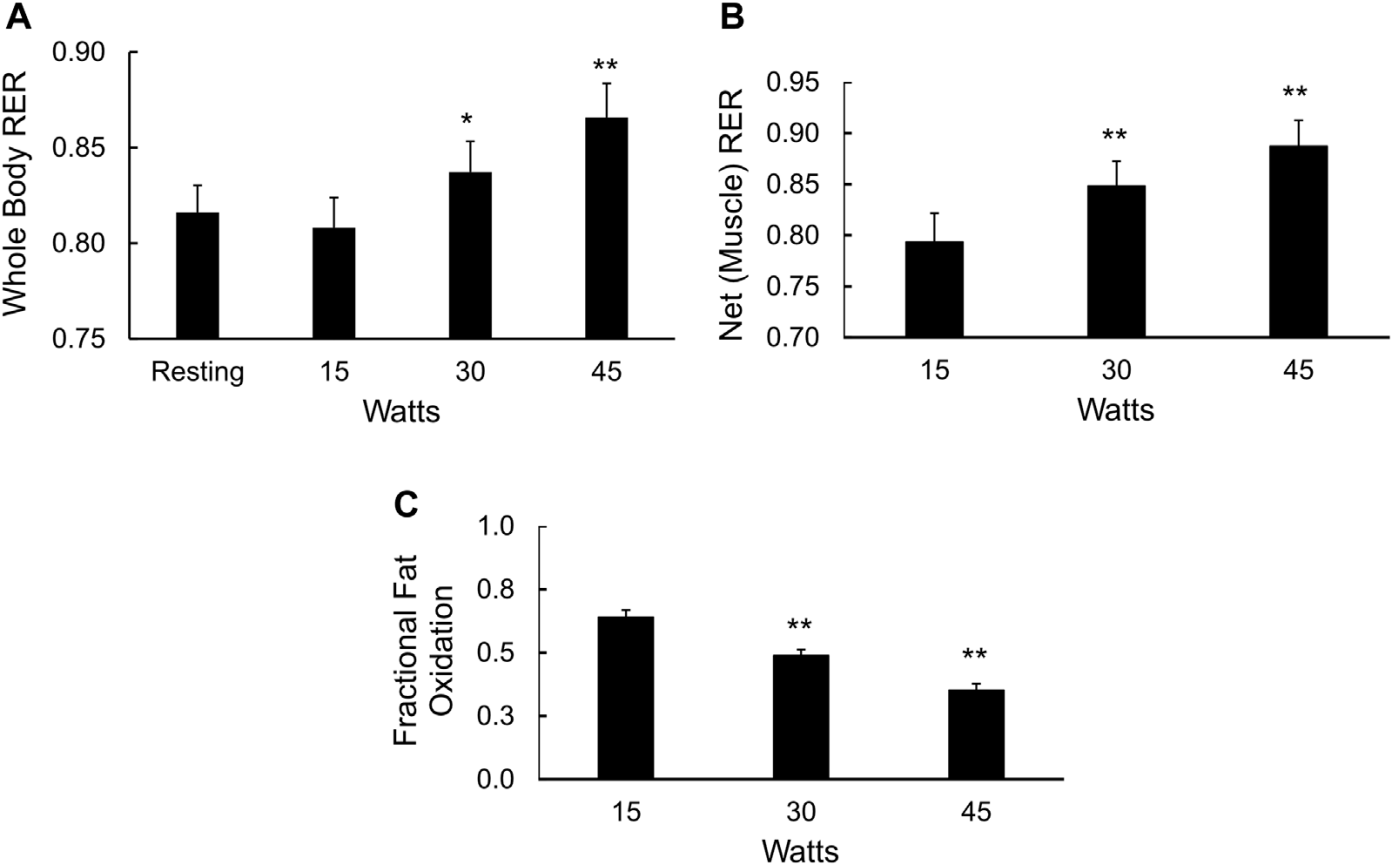
Fuel selection in mildly exercising skeletal muscle. **(A)** Whole body RER at rest and during 15, 30, and 45 W of cycle ergometry exercise, *p < 0.05, **p < 0.01 vs. resting values; **(B)** Net, or working muscle RER (see methods) at 15, 30, and 45 W of cycle ergometry exercise, **p < 0.01 vs. net RER at 15 W; and **(C)** Fractional fat oxidation in working muscle calculated from the delta RER values, **p < 0.01 vs. fractional fat oxidation in working muscle at 15 W.

Cycle ergometer exercise with indirect calorimetry consisted of a period of rest, transition to steady state exercise at 15, 30, and 45 W for 6 min each, followed continuously by a ramp protocol to determine VO_2max_ Figure 4. The respiratory exchange ratio (RER) due to working muscle, or ΔRER (Ganley et al., 2011; Barakati et al., 2022), was calculated using changes in VO_2_ and VCO_2_ from rest to exercise (15 W) and at sequential power outputs of 30 and 45 W. The rise in whole-body RER with increasing power outputs (Figure 4A) was mirrored by the increase in muscle RER (Figure 4B) and a fall in the fraction of total muscle energy expenditure from fat (*p* < 0.01, Figure 4C). The average fraction of energy expenditure derived from fat (fractional fat oxidation during mild exercise) over all three power outputs was 49% ± 2%.

### Multiple linear regression analysis reveals that mitochondrial functional content estimated by the slope of the force-flow relationship and VO_2max_ both are significant, independent predictors of insulin sensitivity

We used a stepwise multiple linear regression approach to assess independent predictors of insulin sensitivity, where the least significant independent variable was removed step-by-step for insulin-stimulated glucose disposal as the dependent variable. For the model in which insulin-stimulated Rd was the dependent variable, we used VO_2max_, total mitochondrial protein abundance, fractional fat oxidation, *τ*, and the force-flow slope as independent variables in the stepwise analysis. For fractional fat oxidation as the dependent variable, we used VO_2max_, total mitochondrial protein abundance, insulin-stimulated Rd, *τ*, and the force-flow slope as independent variables. The rationale for including these variables was that VO_2max_ principally reflects systemic processes including cardiorespiratory variables and blood hemoglobin concentrations (Lundby et al., 2017), mitochondrial protein abundance reflects the protein content of mitochondria, the force-flow slope of ΔG_ATP_ vs. J_ATP_ reflects the *functional* content of muscle mitochondria estimated from ^31^P-MRS, and *τ* is a simply-measured variable related to the rate of oxidative phosphorylation that does not require calibration of energy phosphate or creatine concentrations. Table 3 shows the correlation matrix for all these variables. We report results for the full regression model as well as the final model with stepwise removal of the least significant independent variable.

**TABLE 3:** Pearson correlation coefficients among variables used in multiple regression analyses.

	Rd	VO_2max_	Mito proteins	Fat Fxn	τ	Force-flow slope
Rd	1					
VO_2max_	0.598**	1				
Mito proteins	0.417	0.815**	1			
Fat Fxn	0.158	0.646**	0.739**	1		
τ	−0.367	−0.541*	−0.694**	−0.530*	1	
Force-Flow slope	0.474*	0.101	0.343	0.143	−0.420	1

Rd, insulin-stimulated glucose disposal; VO_2max_, maximal aerobic capacity; Mito proteins, proteomics based total mitochondrial protein abundance; Fat Fxn, proportion of fuel oxidation from lipid during mild exercise; Force-flow slope, slope of the relationship between GATP, and J_ATP_, the rate of oxidative phosphorylation. *p < 0.05, **p < 0.01.

**FIGURE 5:**
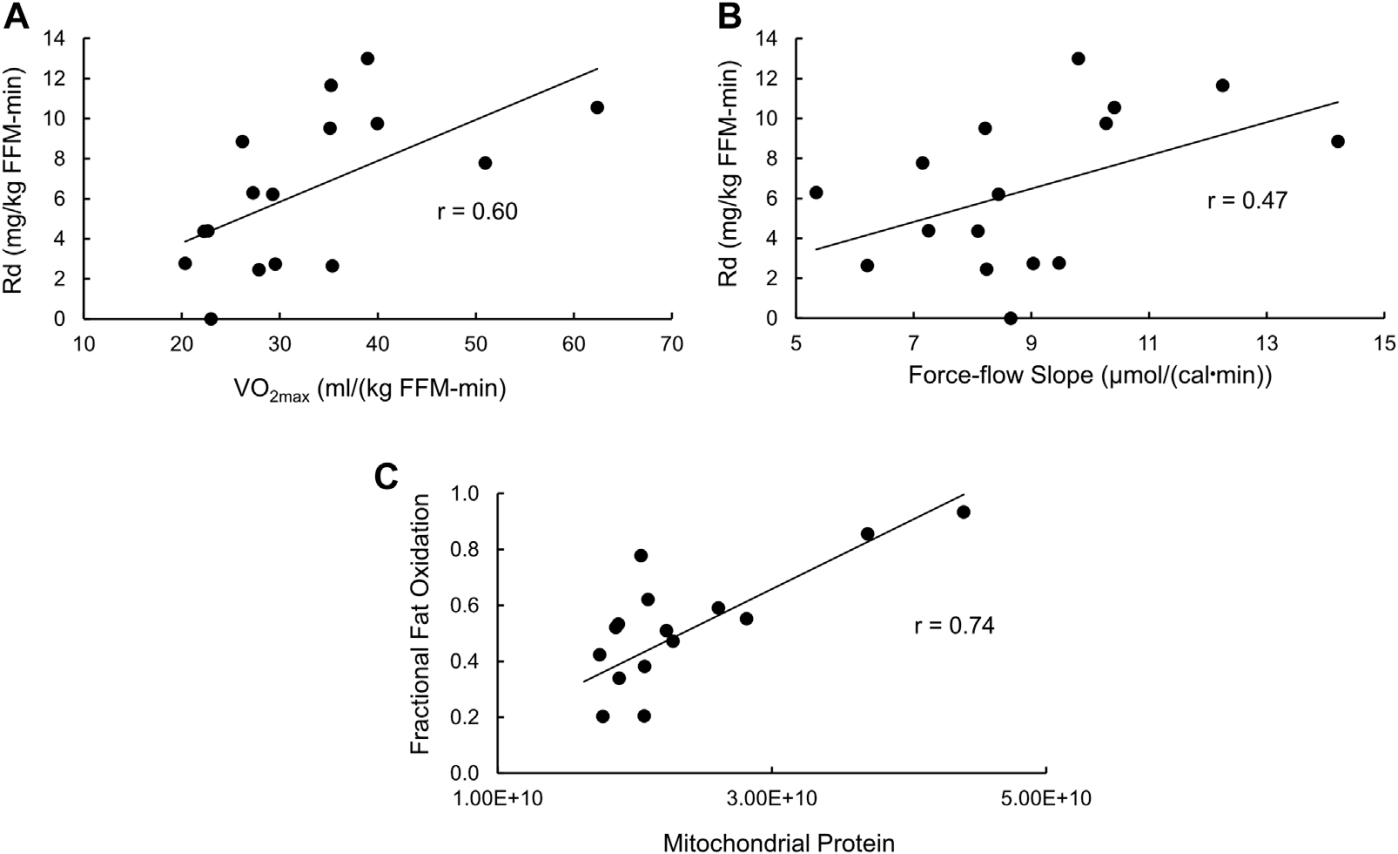
Bivariate relationships between variables that were significant by stepwise multiple regression analyses with either insulin sensitivity or fractional fat oxidation. **(A)** Insulin stimulated glucose disposal (Rd) vs. VO_2max_; **(B)** Rd vs the force-flow slope [conductance or mitochondrial functional content; and **(C)**] fractional fat oxidation during exercise vs. mitochondrial functional content determined using proteomics analysis.

With regard to insulin-stimulated Rd as a measure of insulin action (Supplementary Table S2), the full model has an R^2^ = 0.69 (*p* = 0.02), with only VO_2max_ (*p* = 0.0064) and the force-flow slope (*p* = 0.023) being statistically significant independent variables. Stepwise regression removing the least significant independent variable resulted in a final model with an R^2^ = 0.54 (*p* = 0.0073) and again VO_2max_ (*p* = 0.012) and the force-flow slope (*p* = 0.048) being the only remaining statistically significant independent variables. The simple relationships of insulin-stimulated Rd with VO_2max_ and the force-flow slope are illustrated in Figures 5A, B, respectively. Best subsets multiple regression analysis confirmed this final stepwise model was the best fit.

### Mitochondrial protein abundance is a significant independent predictor of fractional fat oxidation during mild exercise

As for fractional fat oxidation, the R^2^ for the full model was 0.61 (*p* = 0.056), with no independent variable being statistically significant. However, stepwise multiple regression removing the least significant independent variable resulted in a final model with an R^2^ = 0.55 (*p* = 0.001, Supplementary Table S2) and only mitochondrial protein abundance remaining as a statistically significant independent variable (*p* = 0.001). The simple Pearson’s correlation between these variables is shown in Figure 5C.

### Relationship between mitochondrial protein abundance measured by proteomics and citrate synthase (CS) activity

Because CS activity is widely used as a measure of mitochondrial protein content, we compared this measure with the proteomics-determined global mitochondrial protein index. CS activity was 4.45 ± 1.59 μmol·min·g wet weight. CS activity was not correlated with the proteomics-based mitochondrial protein index (*r* = 0.12) but was highly correlated with CS protein abundance (normalized peak area) determined by proteomics (*r* = 0.84, *p* < 0.001). CS activity was not correlated with the fraction of lipid oxidized during mild exercise (*r* = −0.01). Interestingly, CS activity was correlated with the slope of the ΔG_ATP_:J_ATP_ relationship.

## Discussion

Because skeletal muscle insulin resistance often precedes the development of beta cell failure and type 2 diabetes mellitus, the underlying causes of insulin resistance have been the subject of intense interest. Many investigators have used a molecular approach to try to discover the biochemical and molecular factors that lead to insulin resistance. Such studies, many of which have been performed in humans, have provided valuable information about differences in insulin signal transduction, regulation of glucose transport, hexokinase and glucose uptake, glycogen synthesis, mitochondrial function, and gene expression and protein abundance measures (Kahn and Cushman, 1985; Mandarino et al., 1990; Mandarino et al., 1995; Shulman, 1999; Cusi et al., 2000; Kelley and Mandarino, 2000; Petersen and Shulman, 2002; Patti et al., 2003). Many other studies have used genetic manipulation of rodents, usually at a single gene level, to create models of altered insulin action (Wasserman and Ayala, 2005). All of these studies are predicated on the idea that by understanding the molecular details of a system, properties of the whole system can be deduced. However, there are alternate ways of understanding a system that start with fundamental principles of physiology, such as the laws of thermodynamics, and attempt to describe the system based on these principles rather than, or in accompaniment with, a description of biochemical or molecular processes. Both molecular and physiological approaches have value and should be interpreted together, yet to our knowledge, nearly all studies of the origins of skeletal muscle insulin resistance mainly have used a molecular or biochemical approach. In the present study, we asked the question of whether basic thermodynamic principles (nonequilibrium thermodynamics) can be used to explain variance in insulin sensitivity and changes in skeletal muscle fuel preference. To accomplish this, we used calibrated ^31^P-MRS to assess thermodynamic variables in skeletal muscle at rest and during recovery from mild exercise coupled with euglycemic, hyperinsulinemic clamps to assess insulin sensitivity and graded exercise tests to determine fuel selection in working muscle. The participants in the present study purposely were selected to provide a wide range of body composition, insulin action and exercise characteristics. We chose this approach in order to provide the best opportunity to determine relationships between these characteristics and the thermodynamic and fuel selection variables that we measured. Although selection of discrete groups of participants is commonly used in such studies, often the selection of discrete groups can mask the fact that these are continuous variables and participants fall along a continuous spectrum. Our approach in fact resulted in the revelation of novel relationships.

We used “calibrated” ^31^P-MRS to accomplish the purposes of this study (Kemp et al., 2007). Concentrations of energy phosphates that we calibrated to resting [ATP] assayed biochemically in skeletal muscle biopsies for each participant corresponded well to values previously reported using biopsies or ^31^P-MRS (Kemp et al., 2007; Lanza et al., 2011; Ripley et al., 2018). This also was the case for total creatine assayed in biopsies. Both resting [ATP] and total creatine were slightly lower than the often used “standard” assumptions of 8.2 mM [ATP] and 42.5 mM TCr in resting human muscle (Kemp et al., 2007; Kemp et al., 2015; Meyerspeer et al., 2020). What was most important regarding these findings, though, was that resting [ATP] varied from 5.33–8.2 mM and TCr ranged from 24.1–49.7 mM. Without calibration, this high level of variability would have had considerable impact on the calculation of individual values for energy phosphates, total creatine, J_ATP_, and ΔG_ATP_. Errors produced in these variables by using standard assumptions or other constant values likely would have led to an inability to determine whether thermodynamic considerations were related to insulin sensitivity or fuel selection. The importance of using calibrated values has been emphasized by Kemp and coworkers (2007, 2015) and more recently has been explored by Ripley and colleagues, who used a phosphate phantom and the water signal to calibrate energy phosphates and total creatine, respectively (Ripley et al., 2018).

We used a weighted bag system (Sleigh et al., 2016) to drive PCr down by 15%–25% and followed recovery of [PCr] to determine tau and estimate the rate of oxidative phosphorylation (J_ATP_). Along with the corresponding energy state of the cell (ΔG_ATP_). During the two exercise periods [ATP] remained nearly constant while [PCR] fell and [Pi] rose in a nearly equimolar fashion. The time course of [PCr] recovery, which is a function of the rate of oxidative phosphorylation (J_ATP_), is characterized by the time constant τ of a monoexponential function (Meyerspeer et al., 2020). The value of τ was around 30 s in the current study, similar to what has been reported (Conley et al., 2000; Schrauwen-Hinderling et al., 2007a; Lanza et al., 2011; Cuthbertson et al., 2014), and was reproducible, with a coefficient of variation of around 13%. Since τ is estimated independently of calibrated values for energy phosphates or creatine, it is a commonly measured variable and provides some insight into muscle oxidative characteristics. However, in the absence of independent estimates of mitochondrial conductance or muscle capacitance (TCr), the value of τ potentially is misleading (see Supplementary Material for a more detailed discussion of this point). Thus, calibration of MRS signals is essential to assess the role nonequilibrium thermodynamics might play in insulin sensitivity or fuel selection.

To test the hypotheses regarding the relationship between the thermodynamic properties of muscle and insulin sensitivity or fuel selection, it is critical that our data conform to the linear model of nonequilibrium thermodynamics (Meyer, 1989). We showed that both the ΔG_ATP_:J_ATP_ relationship and the relationship between the slope of that relationship and apparent conductance of the linear model (TCr/τ) were highly linear. In addition, the relationship between TCr measured in biopsies and apparent capacitance of the model also was linear and supports the concept that TCr is proportional to the capacitance of the electrical circuit analog model under non-steady state conditions of ΔG_ATP_, such as during recovery from exercise.

We have conjectured that, on a purely theoretical basis, the ability of muscle to maintain a high ΔG_ATP_ in the face of a rise in energy demand could positively influence the rates of insulin-stimulated energy-consuming reactions (Mandarino and Willis, 2023). Supporting this notion, a rise in plasma insulin concentration increases oxygen consumption across the leg under resting conditions (Kelley et al., 1990; Kelley and Mandarino, 1990; Kelley et al., 1992; Kelley et al., 1993; Mandarino et al., 1996), indicating that insulin action induces a mild increase in energy demand. If lower mitochondrial content requires a lower ΔG_ATP_ to meet energy demand, energy demand might be met at the expense of slower rates of insulin-stimulated reactions (Mandarino and Willis, 2023). For example, force production and power output by contractile proteins decline with ΔG_ATP_ (Jeneson et al., 1995; Westerhoff et al., 1995). To the extent that motor proteins may share similar characteristics with proteins like kinesins (Vale and Milligan, 2000), we speculated that the rate of transit of GLUT4 vesicles along cytoskeletal tracks could be sensitive to ΔG_ATP_. We used a simple linear nonequilibrium thermodynamic model to confirm that high conductance in the mitochondrial oxidative pathway predicts higher insulin sensitivity. This could indicate that higher mitochondrial functional content predicts insulin sensitivity. This finding is consistent with the concept that a higher energetic state of muscle may drive one or more insulin-stimulated energy-requiring processes at a faster rate, leading to higher insulin-stimulated glucose disposal during a euglycemic clamp. The current results do not provide direct proof of this idea, but the results provide a compelling argument to pursue additional exploration of this hypothesis. These findings appear to be related to the results of Koch and Britton (2022) that rats selected for running capability are healthier, leaner, more insulin sensitive, and long-lived than rats selected for poor running ability.

The second purpose of this study was to determine if the ability of mitochondria to maintain ΔG_ATP_ in skeletal muscle dictates fuel selection during mild exercise. A lower ΔG_ATP_ would be predicted to lead to higher carbohydrate oxidation at the expense of fat oxidation through elevations in [ADP], [Pi], and potentially [AMP] (Connett, 1989; Stanley and Connett, 1991). Lower ΔG_ATP_ during mild exercise would follow from a lower content of functional mitochondria that lessens the ability of muscle to defend ΔG_ATP_ during exercise or potentially any increase in energy demand (see Supplementary Material). The present results show that mitochondrial protein content is the best linear predictor of the fraction of fuel accounted for by lipid (*r* = 0.74). This is not direct evidence in favor of this mechanism but provides an argument for performing more mechanistic studies in the future to test this hypothesis.

A number of previous studies used ^31^P-MRS *in vivo* in humans to determine the relationship between oxidative phosphorylation defects and insulin sensitivity (Conley et al., 2000; Newcomer et al., 2001; Petersen et al., 2003; Petersen et al., 2004; Schrauwen-Hinderling et al., 2007a; Schrauwen-Hinderling et al., 2007b; Szendroedi et al., 2007; Lanza et al., 2011; Ripley et al., 2018; Toledo et al., 2018). Several studies used the saturation transfer technique (Pi →ATP) to estimate rates of oxidative phosphorylation (Petersen et al., 2003; Petersen et al., 2004; Szendroedi et al., 2007; Kacerovsky-Bielesz et al., 2009). However, this technique does not accurately measure oxidative phosphorylation (Balaban and Koretsky, 2011; From and Ugurbil, 2011; Kemp and Brindle, 2012), leaving those findings open to reinterpretation. Other studies of this topic employing ^31^P-MRS techniques used assumptions, rather than measurements, of the concentrations of ATP and total creatine (TCr) in muscle (Schrauwen-Hinderling et al., 2007a; Schrauwen-Hinderling et al., 2007b). Although these studies, mainly examining recovery of phosphocreatine after exercise, have revealed interesting information regarding the relationships between oxidative phosphorylation and insulin sensitivity, the lack of calibration of energy phosphates or creatine concentrations prohibits their use in determining a role for nonequilibrium thermodynamics in insulin action or fuel selection. Two other studies have used calibrated MRS to understand oxidative phosphorylation changes in aging (Conley et al., 2000) and type 2 diabetes (Ripley et al., 2018). Ripley et al. (2018) used ^31^P-MRS calibrated with a phosphate phantom for energy phosphate concentrations, with quantification of creatine using the water signal, to calculate [ADP] in resting skeletal muscle from insulin resistant obese and type 2 diabetic patients finding no difference in [ADP] between obese and type 2 diabetic volunteers. Rather, these investigators found lower [PCr] to be correlated with several factors including mitochondrial density determined from biopsies. We confirmed this inverse correlation between [PCr] mitochondrial protein content determined using proteomics (*r* = −0.42, *p* < 0.10). The calibrated [ATP] in that study (6–7 mM) was similar to our biopsy-determined value of about 6.8 mM, somewhat below a commonly used “standard” assumption of 8.2 mM. The reasons for these differences in [ATP] are unclear. Unfortunately, neither Conley et al. (2000) nor Ripley et al. (2018) put their findings into the context of nonequilibrium thermodynamics. Nevertheless, the results of Ripley et al. (2018) provide the valuable demonstration that to study mitochondrial function and content *in vivo* in human muscle requires validly calibrated values for concentrations of energy phosphates and total creatine. A unique aspect of the present study is that we used calibrated ^31^P-MRS to study the relationship of metabolic flows to thermodynamic forces and found that functional mitochondrial content and efficient oxidative energy transfer predict insulin sensitivity.

We also used a new label-free proteomics approach to calculate an index of mitochondrial protein content using lysates of whole muscle protein. This simple index was taken to be the sum of all individual abundance levels associated with proteins assigned to mitochondria. Nearly 500 mitochondrial proteins out of a total of over 4000 total proteins were quantified in this analysis. The use of hundreds rather than a single mitochondrial protein as an index of mitochondrial protein content should provide a more accurate measurement of mitochondrial content. However, mitochondrial functional content, measured as conductance in the electrical circuit linear model of nonequilibrium thermodynamics may be a better indicator of mitochondrial function than mere mitochondrial protein content with respect to insulin sensitivity. Even so, mitochondrial protein content was a good predictor of fat oxidation during mild exercise and citrate synthase activity was related to the apparent conductance of the model we used.

In conclusion, the results of the present study show that insulin resistance and a preference for carbohydrate oxidation in skeletal muscle can be explained by a lower free energy state that likely results from lower mitochondrial functional and protein content, as recently conjectured (Mandarino and Willis, 2023). The latter, in turn, may be a consequence of lower physical activity, leading to reduced mitochondrial functional content, although this study does not directly address the question of chronic physical activity. A number of studies have used various aspects of ^31^P-MRS to assess mitochondrial function in healthy and insulin resistant humans, but to our knowledge, this is the first study to use calibrated ^31^P-MRS to assess the energy state of muscle at rest and during mild exercise in the context of insulin resistance in the context of a model of skeletal muscle thermodynamics. These results show that strategies to raise skeletal muscle mitochondrial functional content, whether by increased physical activity, new pharmacological approaches, or even mitochondrial transplant (Guariento et al., 2021) likely would improve both insulin sensitivity and lipid oxidation and potentially reduce the risk of type 2 diabetes and cardiovascular disease.

## Data Availability

The original contributions presented in the study are publicly available. This data can be found here: https://proteomecentral.proteomexchange.org/cgi/GetDataset?ID=pxd043032.
