# Supplementary material for: Nonequilibrium thermodynamics and mitochondrial protein content predict insulin sensitivity and fuel selection during exercise in human skeletal muscle
Source: Front Physiol. 2023 Jul 7;14:1208186. doi: 10.3389/fphys.2023.1208186 (PMC10361819; doi:10.3389/fphys.2023.1208186)
Supplement: Supplementary file 1 [file Table2.DOCX]

**Supplementary Table 2.** Stepwise multiple regression analyses.

| **Full model for insulin-stimulated Rd as dependent variable.** | | | |  |  |  |  |
| --- | --- | --- | --- | --- | --- | --- | --- |
| SUMMARY OUTPUT | |  |  |  |  |  |  |
|  |  |  |  |  |  |  |  |
| *Regression Statistics* | |  |  |  |  |  |  |
| Multiple R | 0.831 |  |  |  |  |  |  |
| R Square | 0.691 |  |  |  |  |  |  |
| Adjusted R Square | 0.536 |  |  |  |  |  |  |
| Standard Error | 2.626 |  |  |  |  |  |  |
| Observations | 16 |  |  |  |  |  |  |
|  |  |  |  |  |  |  |  |
| ANOVA |  |  |  |  |  |  |  |
|  | *df* | *SS* | *MS* | *F* | *Significance* |  |  |
| Regression | 5 | 153.89 | 30.78 | 4.46 | 0.021 |  |  |
| Residual | 10 | 68.94 | 6.89 |  |  |  |  |
| Total | 15 | 222.83 |  |  |  |  |  |
|  |  |  |  |  |  |  |  |
|  | *Coefficients* | *Standard Error* | *t* | *P* | *Lower 95%* | *Upper 95%* | |
| Intercept | -6.624 | 8.519 | -0.778 | 0.455 | -25.605 | 12.357 | |
| VO_2max_ | 0.379 | 0.110 | 3.436 | 0.000 | 0.133 | 0.624 | |
| Mito protein content | 0.000 | 0.000 | -1.121 | 0.288 | 0.000 | 0.000 | |
| Fractional FatOx | -4.743 | 4.173 | -1.137 | 0.282 | -14.041 | 4.555 | |
| τ | -0.010 | 0.165 | -0.063 | 0.951 | -0.377 | 0.356 | |
| Force-Flow slope | 0.978 | 0.366 | 2.673 | 0.023 | 0.163 | 1.794 | |

VO_2max_, maximal oxygen consumption; Mito protein content, mitochondrial protein content determined using proteomics; Fractional FatOx, average fraction of fuel oxidation from fat during mild exercise (15, 30, 45 watts); τ, rate constant for PCr recovery after exercise; Force-Flow slope, slope of the ΔG_ATP_:Jp relationship.

| **Final model for insulin-stimulated Rd as dependent variable.** | | | | | |  |  | |  | |  |
| --- | --- | --- | --- | --- | --- | --- | --- | --- | --- | --- | --- |
| SUMMARY OUTPUT | | |  |  |  | | |  | |  | |
|  |  | |  |  |  | | |  | |  | |
| *Regression Statistics* | | |  |  |  | | |  | |  | |
| Multiple R | 0.728 | |  |  |  | | |  | |  | |
| R Square | 0.530 | |  |  |  | | |  | |  | |
| Adjusted R Square | 0.458 | |  |  |  | | |  | |  | |
| Standard Error | 2.837 | |  |  |  | | |  | |  | |
| Observations | 16 | |  |  |  | | |  | |  | |
|  |  | |  |  |  | | |  | |  | |
| ANOVA |  | |  |  |  | | |  | |  | |
|  | *df* | | *SS* | *MS* | *F* | | | *Significance* | |  | |
| Regression | 2 | | 118.197 | 59.099 | 7.343 | | | 0.007 | |  | |
| Residual | 13 | | 104.632 | 8.049 |  | | |  | |  | |
| Total | 15 | | 222.829 |  |  | | |  | |  | |
|  |  | |  |  |  | | |  | |  | |
|  | | *Coefficients* | *Standard Error* | *t* | *P* | | | *Lower 95%* | | *Upper 95%* | |
| Intercept | | -6.384 | 3.582 | -1.782 | 0.098 | | | -14.122 | | 1.354 | |
| VO_2max_ | | 0.191 | 0.065 | 2.912 | 0.012 | | | 0.049 | | 0.332 | |
| Force-Flow slope | | 0.733 | 0.336 | 2.184 | 0.048 | | | 0.008 | | 1.458 | |

| **Full model for Fractional Fat Oxidation as dependent variable.** | | | | | | |  | |  | |  | | |
| --- | --- | --- | --- | --- | --- | --- | --- | --- | --- | --- | --- | --- | --- |
| SUMMARY OUTPUT | | |  | |  |  | |  | |  | |  |  |
|  |  | |  | |  |  | |  | |  | |  |  |
| *Regression Statistics* | | |  | |  |  | |  | |  | |  |  |
| Multiple R | 0.784 | |  | |  |  | |  | |  | |  |  |
| R Square | 0.614 | |  | |  |  | |  | |  | |  |  |
| Adjusted R Square | 0.422 | |  | |  |  | |  | |  | |  |  |
| Standard Error | 0.187 | |  | |  |  | |  | |  | |  |  |
| Observations | 16.000 | |  | |  |  | |  | |  | |  |  |
|  |  | |  | |  |  | |  | |  | |  |  |
| ANOVA |  | |  | |  |  | |  | |  | |  |  |
|  | *df* | | *SS* | | *MS* | *F* | | *Significance* | |  | |  |  |
| Regression | 5 | | 0.559 | | 0.112 | 3.187 | | 0.056 | |  | |  |  |
| Residual | 10 | | 0.351 | | 0.035 |  | |  | |  | |  |  |
| Total | 15 | | 0.909 | |  |  | |  | |  | |  |  |
|  |  | |  | |  |  | |  | |  | |  |  |
|  | *Coefficients* | | | *Standard Error* | *t* | *P* | | *Lower 95%* | | *Upper 95%* | | |  |
| Intercept | | -0.009 | | 0.626 | -0.014 | 0.989 | | -1.403 | | 1.385 | | |  |
| VO_2max_ | | 0.010 | | 0.011 | 0.939 | 0.370 | | -0.014 | | 0.035 | | |  |
| Mito protein content | | 1.37E-11 | | 1.53E-11 | 0.894 | 0.392 | | -2.05E-11 | | 4.79E-11 | | |  |
| Rd | | -0.024 | | 0.021 | -1.137 | 0.282 | | -0.071 | | 0.023 | | |  |
| τ | | -0.003 | | 0.012 | -0.279 | 0.786 | | -0.029 | | 0.023 | | |  |
| Force-Flow slope | | 0.011 | | 0.034 | 0.318 | 0.757 | | -0.065 | | 0.087 | | |  |

VO_2max_, maximal oxygen consumption; Mito protein content, mitochondrial protein content determined using proteomics; Fractional FatOx, average fraction of fuel oxidation from fat during mild exercise (15, 30, 45 watts); τ, rate constant for PCr recovery after exercise; Force-Flow slope, slope of the ΔG_ATP_:Jp relationship; Rd, insulin-stimulated glucose disposal.

| **Final model for Fractional Fat Oxidation as dependent variable.** | | | | |  | |  | | |  | |
| --- | --- | --- | --- | --- | --- | --- | --- | --- | --- | --- | --- |
| SUMMARY OUTPUT | | |  |  | |  | |  |  |  |  |
|  |  |  |  |  | |  | |  |  |  |  |
| *Regression Statistics* | |  |  |  | |  | |  |  |  |  |
| Multiple R | 0.739 |  |  |  | |  | |  |  |  |  |
| R Square | 0.546 |  |  |  | |  | |  |  |  |  |
| Adjusted R Square | 0.514 |  |  |  | |  | |  |  |  |  |
| Standard Error | 0.172 |  |  |  | |  | |  |  |  |  |
| Observations | 16 |  |  |  | |  | |  |  |  |  |
|  |  |  |  |  | |  | |  |  |  |  |
| ANOVA |  |  |  |  | |  | |  |  |  |  |
|  | *df* | *SS* | *MS* | *F* | | *Significance* | |  |  |  |  |
| Regression | 1 | 0.497 | 0.497 | 16.838 | | 0.001 | |  |  |  |  |
| Residual | 14 | 0.413 | 0.029 |  | |  | |  |  |  |  |
| Total | 15 | 0.909 |  |  | |  | |  |  |  |  |
|  |  |  |  |  | |  | |  |  |  |  |
|  | *Coefficients* | *Standard Error* | *t Stat* | *P-value* | | *Lower 95%* | | *Upper 95%* | | |  |
| Intercept | -0.06629 | 0.143 | -0.463 | 0.650 | | -0.373 | | 0.241 | | |  |
| Mito protein content | 2.42E-11 | 5.89E-12 | 4.103 | 0.001 | | 1.15E-11 | | 3.68E-11 | | |  |
