# Supplementary material for: Nonequilibrium thermodynamics and mitochondrial protein content predict insulin sensitivity and fuel selection during exercise in human skeletal muscle
Source: Front Physiol. 2023 Jul 7;14:1208186. doi: 10.3389/fphys.2023.1208186 (PMC10361819; doi:10.3389/fphys.2023.1208186)
Supplement: Supplementary file 3 [file DataSheet1.docx]

**Supplementary Material**

**Theoretical Considerations and Calculations**

*Free Energy of ATP Hydrolysis (ΔG_ATP_)*

Energy is made available to drive an ATP-linked endergonic process, for example muscle contraction or ion pumping against an electrochemical gradient, when ATP is hydrolyzed to ADP and inorganic phosphate:

(S1) ATP + H_2_O 🡪 ADP + Pi + H^+^

At constant pH we can eliminate the H^+^ when we write the equilibrium constant (K_ATP_):

(S2) $K_{ATP}=\left\lfloor\frac{\left[ ADP \right][Pi]}{[ATP]} \right\rfloor$

The water term can also be neglected since its high concentration (~55 M) does not change with advancement of the reaction. We also must account for the many ionic forms of the participant molecular species, such as chelation by metals, *e.g.,* magnesium. The K_eq_ is determined under roughly physiological conditions, such as free [Mg^2+^] = 1 mM, and all ionic forms are collectively measured as [ΣATP], [ΣADP], and [ΣPi] (Nicholls Bioenergetics 4 (Nicholls and Ferguson, 2013). With such assumptions and at pH 7.0, the K_ATP_ has been measured to be 2.19 x 10^5^ (Golding et al., 1995):

(S3) ΣATP 🡪 ΣADP + ΣPi K_ATP_ = 2.19 x 10^5^ at pH 7.0 and [Mg^+2^]_free_ = 1.0 mM

This extremely large K_eq_ suggests a strong tendency to spontaneously proceed from left to right, but the direction of the reaction arrow, as well as the energy made available as it advances, fundamentally depend on the concentrations of the reactants and products. The ratio of products/reactants is termed the mass action ratio (MAR):

(S4) $MAR= \frac{\left[ \Sigma ADP \right][\Sigma Pi]}{[\Sigma ATP]}$

If the ATP hydrolysis reaction, or any reaction, is allowed to proceed to equilibrium, then the MAR is equal to (indeed, is synonymous with) the K_eq_ and it provides exactly zero driving force to a coupled process. The equation to calculate the Gibbs energy of ATP hydrolysis quantitatively confirms this intuition (Eq S5):

(S5) 1) $\Delta G_{ATP}=RT*Ln\left\lfloor\frac{\frac{\left[ \sum ADP \right]\left[ \sum Pi \right]}{\left[ \sum ATP \right]}}{K_{ATP}} \right\rfloor= RT*Ln\left\lfloor\frac{MAR}{K_{ATP}} \right\rfloor$

where MAR is the mass action ratio, R is the gas constant (1.987 cal deg^-1^ mol^-1^), and T is absolute temperature (310^o^K). To emphasize, if the reaction is at equilibrium, then MAR = K_ATP_, Ln(MAR/K_ATP_) = 0, and ΔG_ATP_ = 0.

*Non-Invasive Assessment of ΔG_ATP_ Using ^31^P-MRS*

The energetic state (ΔG_ATP_) and oxidative phosphorylation rate of muscle can be non-invasively and continuously assessed with ^31^P-MRS methodology (Kemp 2015). As we saw above in Eq S5, the calculation of ΔG_ATP_ requires the molar concentrations of ATP, ADP, and Pi. Spectral peaks for ATP and Pi are visible in ^31^P-MRS, while ADP is not visible (due to the extremely low concentration of free ADP noted above). Muscle water concentrations of [ATP] and [Pi] can be quantified in two ways: 1) Most studies in the literature assume a resting muscle [ATP] of 8.2 mM (as reviewed in Kemp 2007). In this approach the integrated area under the resting ATP spectrum is assumed to be equivalent to 8.2 mM and then proportionality is used to convert all other peak areas to molar concentrations, which includes Phosphocreatine (PCr). 2) The second approach has been termed “calibrated ^31^P-MRS” by Kemp (Kemp et al., 2007). One way to achieve this calibration is to place a phantom standard containing a phosphate-containing molecule of known concentration into the magnet. Alternatively, chemical analysis of muscle biopsies for total adenylate can be used to calibrate the area under the ATP peak, since ATP accounts for >99% of the resting adenylate pool (ATP, ADP, AMP). **In the present studies we calibrated the ^31^P-MRS ATP spectrum by assaying muscle biopsies for ATP using a luciferase-based system.** Prior to the luciferase readout, biopsy samples were pre-treated with a phosphorylating system to ensure near complete conversion of all adenylate into ATP.

Because the ADP peak is not visible, [ADP] is estimated based on the assumption that equilibrium is maintained in the creatine kinase reaction:

(S6) PCr + ADP + H^+^ ⇄ Cr + ATP

(S7) $K_{ck}= \frac{\left[ Cr \right]\left[ ATP \right]}{\left[ PCr \right][ADP]\left[ H^{+} \right]}$

where K_ck_ is 1.66 x 10^9^ M^-1^ (Lawson and Veech, 1979).

Kck is then used to calculate the free ADP concentration:

(S8) $\left[ ADP \right]= \frac{\left[ Cr \right]\left[ ATP \right]}{\left[ PCr \right]\left[ H^{+} \right]K_{ck}}$

Muscle [H^+^] was determined using the chemical shift between PCr and Pi (Walter et al., 1997):

(S9) pH = 6·75 + log(δ − 3·27)/(5·69 − δ)

The assumption that creatine kinase maintains equilibrium at rest and during aerobic exercise has been rigorously validated (Kushmerick, 1998). Notice, however, that Equation S8 includes creatine (Cr), another molecule that is obviously not ^31^P-MRS visible. One calculation of [ADP] assumes that the total creatine pool (TCr = PCr + Cr) of skeletal muscle is 42.5 mM, despite abundant evidence of appreciable variability (Harris et al., 1992). Thus, the present study additionally assayed muscle biopsies for total creatine by chemical analysis after all PCr had been hydrolyzed to Cr by acid pre-conditioning (De Saedeleer and Marechal, 1984)**.**

To summarize, in resting, contracting, and recovering muscle we performed calibrated ^31^P-MRS determinations of [ATP], [Pi], and [PCr] concentrations, and calculated [ADP] based on equilibrium at creatine kinase. [Cr] was calculated by chemically analyzing TCr in biopsies and subtracting [PCr] ([Cr] = TCr – [PCr]). **These assessments provided accurate, non-invasive, and continuous evaluation of ATP free energy and [ADP] calculated using Eq S5 and Eq S8**, respectively. Further, during resting recovery from exercise, **the rate of oxidative phosphorylation (J_ATP_ in mM/min) was estimated from changes in [PCr].** [PCr] across time was also analyzed for the time constant of PCr recovery. The (ΔG_ATP_, J_ATP_) data pairs during recovery were analyzed in the context of nonequilibrium thermodynamics, to determine the conductance of the oxidative phosphorylation pathway, as described below.

*Mitochondrial Driving Forces Generate ΔG_ATP_*

If all three molecular participants in the ATP hydrolysis reaction were maintained at 1.0 molar (M) concentrations, then the MAR would equal 1.0 and Eq 5 shows that ΔG_ATP_ would be equal to (1.987)(310)*Ln[1.0/(2.19*10^5^)] = -7574 cal/mol. In other words, this is simply the definition of the *standard free energy* of the ATP hydrolysis reaction. *In vivo*, however, mitochondrial driving forces in effect “push” Eq S1 from right to left, decreasing [ADP] and [Pi] and increasing [ATP] as shown in outline in scheme (S10):

(S10) ΔG_fuel_ 🡪 ΔG_redox_ 🡪 ΔG_H_^+^ 🡪 ΔG_ATP_

Briefly, the chemical energy of oxidized substrates (fuels) sequentially drives the development of the matrix redox potential, hence protonmotive force (ΔG_H_^+^), which in turn drives matrix synthesis of ATP and its export to the cytosol. As a result, in resting human mixed skeletal muscle cells rough estimates of the cytosolic concentrations of ATP, ADP, and Pi are, respectively, 8 mM, 15 μM, and 3 mM (Kemp et al., 2015). Inserting these values (molar concentrations) into Equation S4 gives:

MAR = [(1.5x10^-5^)*(3x10^-3^)/(8x10^-3^)] = 5.63x10^-6^

We can see that in resting human muscle cells mitochondrial chemiosmotic forces drive reaction (S1) from right to left, moving it 11 orders of magnitude away from equilibrium i.e., MAR/K_ATP_ = (5.63x10^-6^)/(2.19x10^5^) = 2.57x10^-11^. Thus, the actual energy made available when ATP is hydrolyzed under resting in vivo conditions is roughly:

(S11) ΔG_ATP_ = (1.987)(310)*LN[(5.63x10^-6^)/(2.19*10^5^)] = -15021 cal/mol

The robust upstream mitochondrial driving forces shown in scheme (S10) have two major functional consequences on the energy metabolism of skeletal muscle cells: 1) Maintenance of lower [ADP] and [Pi] and higher ATP/ADP ratio, which are all strong modulators of glycolytic enzymes (Stanley and Connett, 1991) and 2) stronger (more negative) ΔG_ATP_, which critically affects actin-myosin force production (Pate et al., 1998) and the ion gradients across the sarcolemma (Masuda et al., 1990) and sarcoplasmic reticulum membrane (Hasselbalch and Oetliker, 1983).

*Physiologic Range of Cytosolic ΔG_ATP_ in Skeletal Muscle*

Cytosolic ΔG_ATP_ in resting skeletal muscle cells is roughly -15 kcal/mol (-63 kJ/mol) and falls (“relaxes”) to about -12 kcal/mol (-50 kJ/mol) as exercise intensity approaches the aerobic maximum, or VO_2_max (Jeneson et al., 2009). Over much of this ~3 kcal/mol region of respiratory control, each incremental rise in steady state exercise intensity elicits a fall in ΔG_ATP_ sufficient to stimulate the mitochondrial ATP production rate (J_ATP_) to match the ATP demand. In fact, J_ATP_ (along with O_2_ consumption rate) rises in a near-linear fashion with the fall in ΔG_ATP_, which reflects thermodynamic control of oxidative phosphorylation.

*Nonequilibrium Thermodynamics: Linear Force-Flow Control of Oxidative Phosphorylation*

The near linear relationship between the steady state ΔG_ATP_ and J_ATP_ has been recognized for several decades as one example of nonequilibrium thermodynamic (NET) control of metabolism (Rottenberg 1973; Van der Meer et al. 1980; Westerhoff et al. 1981; Westerhoff and VanDam, 1987; Davis and Davis-Van Thienen 1989). NET rate equations are analogous to Ohm’s Law, where net metabolic flux (current) is the consequence of a thermodynamic driving force (voltage drop) times a phenomenological conductance (conductance = 1/resistance).

(S12) Voltage = Current * Resistance (Ohm’s Law)

Rearranged, Current = (Voltage drop)/Resistance = (Voltage drop) * Conductance

Analogous form, Flow = (Net Driving Force) * Conductance

where metabolic flow is ATP production rate and conductance is proportional to mitochondrial oxidative pathway enzyme abundance and activation state. The net driving force increases as ΔG_ATP_ falls, see Scheme S10.

For example, in vitro mitochondrial O_2_ consumption rate can be measured with an O_2_ electrode while the bathing medium ΔG_ATP_ is experimentally manipulated with a “PCr energy clamp” procedure (Willis et al., 2016). ATP production and O_2_ consumption rise to higher steady state rates as ΔG_ATP_ is “relaxed” stepwise to less negative values (decreased medium PCr/Cr ratios). The relationship is linear, yielding a J_ATP_/ΔG_ATP_ slope which evaluates the conductance of the mitochondria in the respiratory chamber. If the same titration of energy state is carried out but with twice as much mitochondrial protein added to the chamber, then the observed slope will also be doubled, reflecting the additional mitochondrial conductance (Glancy et al., 2008). Moreover, if the oxidative pathway is analyzed in segments as shown above in scheme (S10), the flux of each segment varies linearly with the free energy difference (ΔΔG) down that segment (Glancy et al., 2013). Near-linear ΔG_ATP_:J_ATP_ control is also non-invasively observed *in vivo* in human muscle using ^31^P-MRS (Westerhoff et al., 1995;Jeneson et al., 2009).

To summarize linear force-flow, ΔG_ATP_:J_ATP_, control, we once again refer to scheme (S10): In healthy muscle, mitochondria generate the “upstream” driving forces, which are transferred to ΔG_ATP_ down an abundant, high-conductance oxidative pathway. At rest, muscle ΔG_ATP_ is high (robust) and this strong “backpressure” results in a low net flux from left to right, as intuition would predict. When the muscle transitions from rest to exercise, the higher rate of ATP breakdown “relaxes” the downstream ΔG_ATP_ to a less negative value and flux toward ATP synthesis increases in a first order fashion until it matches the new rate of ATP demand. At a given ATP demand, the magnitude of the fall in steady state ΔG_ATP_ depends on the conductance term (Eq S12). High mitochondrial content provides a high conductance oxidative pathway, thus better defense of cytosolic energy state. Better defense of ΔG_ATP_ predicts glycolytic pathway suppression (Stanley and Connett, 1991), thus greater fat oxidation, and also a stronger driving force delivered to ATP-utilizing sites.

*NET during a Metabolic Transient*

In 1988 Meyer (Meyer, 1988) confirmed and extended the validity of the thermodynamic control of oxidative phosphorylation by including the concept of metabolic capacitance (Sweeney, 1994). Meyer’s model explains control of steady state ATP turnover exactly as the NET model described above, but it it also accounts for the time course of the J_ATP_ adjustment to a new demand during a metabolic transient. Meyer modeled oxidative phosphorylation control as an RC electrical circuit, in which “R” is mitochondrial resistance (as above) and “C” is capacitance, which biologically is primarily due to the presence of the creatine kinase reaction and the high concentration of total creatine (TCr = PCr + creatine) in skeletal muscle. When ATP demand changes from one steady state J_ATP_ to another, the period of adjustment is described by a first order time constant called tau (τ). The value of tau is predicted by:

(S13) τ = R * C

where “R” is mitochondrial resistance to energy transfer (identical to the definition above) and “C” is metabolic capacitance, which is proportional to muscle TCr. If, as before, we use the reciprocal of resistance i.e., conductance, then:

(S14) τ = Capacitance/Conductance

and rearranged:

(S15) Capacitance = τ * Conductance

(S16) Conductance = Capacitance/τ

In practical terms it is obvious from Equation S14 that fast adjustment (small τ) from one steady state J_ATP_ to another can be achieved with either high conductance (high mitochondrial abundance) or small capacitance (low TCr) or some combination of the two factors. We used Equations S15 and S16 as internal checks on the measurements made in the present study.

*Experimental Estimates of Metabolic Capacitance and Mitochondrial Conductance*

Muscle TCr was chemically assayed in muscle biopsies to both estimate metabolic capacitance and to calibrate the analysis of ^31^P-MRS data, as described above. Mitochondrial conductance was evaluated as the slope of (ΔG_ATP_, J_ATP_) data pairs obtained during recovery from mild-to-moderate exercise. Two minutes of knee extension exercise in the magnet (19.9 ± 7.7% net breakdown of rest [PCr]) was followed by four minutes of recovery. During the first 1.0 to 1.5 minute of recovery, the instantaneous rate of ATP production, J_ATP_, was estimated from the change in [PCr] at each 7.5 sec time point, along with the corresponding ΔG_ATP_, calculated as described above. The (ΔG_ATP_, J_ATP_) data pairs were then used to plot J_ATP_ as a function of ΔG_ATP_. The slope of these linear ΔG_ATP_:J_ATP_ relationships is the conductance of the oxidative phosphorylation pathway described above. If this methodological approach is valid, then there should be agreement between this ΔG_ATP_:J_ATP_ force-flow slope estimate of conductance and the ratio TCr/τ, as predicted by Equation S16. As reported in the paper, these two estimates did in fact correlate very highly, r = 0.73. Similarly, there was excellent agreement, r = 0.77, between TCr vs. the product of tau and the force-flow slope, as predicted by Equation S15. These methodological checks lend support to both the validity of the experimental model and the accuracy of the measurements. Further, these data emphasize the benefit of calibrated ^31^P-MRS analysis. Obviously, if one value of TCr had been assumed for all subjects, for example the 42.5 mM often used conventionally, then Equations S14, S15, and S16 would have no meaning. Moreover, these findings caution against the interpretation of τ measured in isolation. For example, if TCr levels are appreciably heterogeneous in a group, but are not measured and assumed equal, then particularly high or low τ values may simply reflect, respectively, low or high TCr, and have little or no implications regarding mitochondrial functional status.

**Supplementary References**

De Saedeleer, M., and Marechal, G. (1984). Chemical energy usage during isometric twitches of frog sartorius muscle intoxicated with an isomer of creatine, beta-guanidinopropionate. *Pflugers Arch* 402**,** 185-189.

Glancy, B., Willis, W.T., Chess, D.J., and Balaban, R.S. (2013). Effect of calcium on the oxidative phosphorylation cascade in skeletal muscle mitochondria. *Biochemistry* 52**,** 2793-2809.

Golding, E.M., Teague, W.E., Jr., and Dobson, G.P. (1995). Adjustment of K' to varying pH and pMg for the creatine kinase, adenylate kinase and ATP hydrolysis equilibria permitting quantitative bioenergetic assessment. *J Exp Biol* 198**,** 1775-1782.

Harris, R.C., Soderlund, K., and Hultman, E. (1992). Elevation of creatine in resting and exercised muscle of normal subjects by creatine supplementation. *Clin Sci (Lond)* 83**,** 367-374.

Jeneson, J.A., Schmitz, J.P., Van Den Broek, N.M., Van Riel, N.A., Hilbers, P.A., Nicolay, K., and Prompers, J.J. (2009). Magnitude and control of mitochondrial sensitivity to ADP. *Am J Physiol Endocrinol Metab* 297**,** E774-784.

Kemp, G.J., Ahmad, R.E., Nicolay, K., and Prompers, J.J. (2015). Quantification of skeletal muscle mitochondrial function by 31P magnetic resonance spectroscopy techniques: a quantitative review. *Acta Physiol (Oxf)* 213**,** 107-144.

Kemp, G.J., Meyerspeer, M., and Moser, E. (2007). Absolute quantification of phosphorus metabolite concentrations in human muscle in vivo by 31P MRS: a quantitative review. *NMR Biomed* 20**,** 555-565.

Kushmerick, M.J. (1998). Energy balance in muscle activity: simulations of ATPase coupled to oxidative phosphorylation and to creatine kinase. *Comp Biochem Physiol B Biochem Mol Biol* 120**,** 109-123.

Lawson, J.W., and Veech, R.L. (1979). Effects of pH and free Mg2+ on the Keq of the creatine kinase reaction and other phosphate hydrolyses and phosphate transfer reactions. *J Biol Chem* 254**,** 6528-6537.

Masuda, T., Dobson, G.P., and Veech, R.L. (1990). The Gibbs-Donnan near-equilibrium system of heart. *J Biol Chem* 265**,** 20321-20334.

Meyer, R.A. (1988). A linear model of muscle respiration explains monoexponential phosphocreatine changes. *Am J Physiol* 254**,** C548-553.

Nicholls, D.G., and Ferguson, S.J. (2013). *Bioenergetics.* Boston: Elsevier.

Pate, E., Franks-Skiba, K., and Cooke, R. (1998). Depletion of phosphate in active muscle fibers probes actomyosin states within the powerstroke. *Biophys J* 74**,** 369-380.

Stanley, W.C., and Connett, R.J. (1991). Regulation of muscle carbohydrate metabolism during exercise. *FASEB J* 5**,** 2155-2159.

Sweeney, H.L. (1994). The importance of the creatine kinase reaction: the concept of metabolic capacitance. *Med Sci Sports Exerc* 26**,** 30-36.

Walter, G., Vandenborne, K., Mccully, K.K., and Leigh, J.S. (1997). Noninvasive measurement of phosphocreatine recovery kinetics in single human muscles. *Am J Physiol* 272**,** C525-534.

Westerhoff, H.V., Van Echteld, C.J., and Jeneson, J.A. (1995). On the expected relationship between Gibbs energy of ATP hydrolysis and muscle performance. *Biophys Chem* 54**,** 137-142.

Willis, W.T., Jackman, M.R., Messer, J.I., Kuzmiak-Glancy, S., and Glancy, B. (2016). A Simple Hydraulic Analog Model of Oxidative Phosphorylation. *Med Sci Sports Exerc* 48**,** 990-1000.
